# Influence of public hospital reform on public health: Evidence from a quasi-natural experiment in China

**DOI:** 10.3389/fpubh.2023.1104328

**Published:** 2023-03-24

**Authors:** Bingnan Guo, Weizhe Feng, Huilin Cai, Ji Lin

**Affiliations:** ^1^School of Humanities and Social Sciences, Jiangsu University of Science and Technology, Zhenjiang, China; ^2^School of Marxism, Jiangnan University, Wuxi, China; ^3^School of Economics and Management, Wenzhou University of Technology, Wenzhou, China

**Keywords:** public hospital reform, public health, medical expenditure, DID, the mediating effect, China

## Abstract

Public health is an important symbol of national wealth and prosperity. At present, China's public health is hindered by the poor management of public hospitals, which impacts the demographic structure and socioeconomic development. Therefore, taking the implementation of public hospital reform in China as a quasi-natural experiment, this study employed the time-varying DID model and the mediating effect to evaluate the influence of public hospital reform on public health. The results were as follows: (1) Public hospital reform can significantly improve public health, and a series of robustness tests have also confirmed the effects; (2) Government's financial support is a transmission mechanism for public hospital reform to promote public health; (3) After taking control variables into consideration, the effect of public hospital reform is stronger in the western region with a poorer economy. This research provides a vital policy reference for promoting the scope of reform and improving the health of the general public.

## 1. Introduction

Since the reform and opening up, China's economy has achieved significant growth, which has been recognized as a miracle in the history of the world economy (1). However, compared to the speed of economic development, the health level of Chinese people has been relatively slow. The development of the health sector has not been satisfactory. In response to the increasingly serious problems in public health, China launched a new round of healthcare reform in 2009 which not only changed the previous market-oriented and commercialized health service but also enhanced the government's responsibility in the health sector.

The disparity between economic growth and health improvement has plagued China for a long time (2). During the early years of reform and opening up, China's mortality rate was effectively decreased through the improvement of basic living and health conditions, but the overall health status was still slow compared to other countries. In terms of life expectancy, for instance, it had increased in low-, middle-, and high-income countries by three, five, and 4 years, respectively, while it had only increased by 2 years in China (3). In comparison, in India, where the economy is growing as rapidly as China, its infant mortality rate is much lower than that in China (4). At the same time, China's health status has become more polarized within the population, where the low-birth-weight rate in 2000 for children under 5 years was 3% in urban areas but six times more in rural areas. Since 2005, when the State Council issued the report “The Failure of Old Health Care Reform,” the subject of healthcare reform has become the focus of national attention. In addition, the new round of public hospital reform that was launched in 2009 has raised the problems in the national health system to an unprecedented level.

As an essential spatial carrier to implement the strategy of *Health China*, hospitals are a vital node for improving the quality of public health. In China, public hospitals are the leading providers of health services, which significantly affects the direction of the overall health sector and determines the quality of its public health. However, at the beginning of the 21st century, the government's “decentralization” in the healthcare sector led to the collapse of the publicly funded health system which was established at the beginning of the new China. In the face of continuous reduction of medical financial support, public hospitals began to shift toward the profit-seeking path of supply-side induced demand, thus resulting in “difficult and costly medical treatment,” creating tension between doctors and patients and other chaotic phenomena that endanger China's healthcare. In response, the Ministry of Health issued the “*Guiding Opinions on the Reform of Public Hospital*” in 2010, which pointed out that public hospitals should adhere to national welfare, put the protection of people's health rights first, and encourage scientific development to meet the health needs of the general public. As the core policy of the “new medical reform,” the public hospital reform aims to help accelerate the formation of a medical service system that will be characterized by “serving the people” and achieving the coordination of public health and medical services. Based on this, this study considered the fact that the public hospital reform has been carried out from pilot projects to full-scale implementation as a quasi-natural experiment, explored the impact of public hospital reform on public health, and attempted to answer the following questions: Has public hospital reform significantly improved public health in China? What is the transmission mechanism? Are there any significant differences in the effects of reform between different regions?

## 2. Literature review

Public health is a significant symbol of national wealth and prosperity. Since the 1950's, health issues, as a pivotal component of human capital, have drawn increasing attention and discussion from economists. After Grossman (5) groundbreaking publication “*On the Concept of Health Capital and the Demand for Health*,” the field of health economics has rapidly developed.

At present, academic research on health economics mainly focuses on the influential factors and the character of its public goods. Among them, the influential factors can be divided into individual and social dimensions. In the individual dimension, factors such as family status, living habits, and medical conditions are strongly related to their health level. In particular, the relative deprivation of income, job, or other economic factors hinders the low-income groups' ability to integrate into society resulting in their low health level (6). Currie et al. (7) believed that people with higher socioeconomic status have more convenient opportunities to enjoy a healthy lifestyle because of the “privilege” of obtaining more health support. In addition, an increase in educational attainment can also lead to a higher health status (8), and Mao et al. (9) found that education can contribute to people's health through a combination of mediating and moderating effects. Regarding the social dimension, scholars have mainly studied it from macro perspectives, such as economic growth, level of openness, environmental pollution, urbanization progress, and scientific improvement. Cen et al. (10) argued that economic growth would have a positive impact on public health due to the presence of government incentives for livelihood. The level of openness will promote public health by bringing healthy food and medical equipment to the residents (11). The significant effect of urbanization, an essential modern civilization benchmark, on improving public health has regional differences (12). As for the effect of environmental pollution, Sun et al. (13) used a spatial econometric model to analyze air pollution with a spillover character and found that it has a more significant detrimental effect on public health than other pollutants (14).

The great positive externality of public health determines that its provision must involve the participation of the public sector. Researchers believe that the government must take responsibility for public health investment and fully play the role of public finance in health investment (15). Medical expenditure can improve the equity of healthcare resource allocation, effectively prolong life span and reduce infant mortality (16). Cheng et al. (17) concluded that medical expenditure not only directly improves people's health status but also moderates the health effects of education. This view is further corroborated by Tian et al. (18) from the perspective of government competition. However, several scholars are skeptical of this conclusion, arguing that health fiscal spending does not have a significant effect on health (19) and even leads to an increase in population mortality (20).

Public hospital reform is the core of China's “new medical reform,” and scholars have mainly focused on the two aspects of the practices and effects. For one, public hospital reform should establish a robust accountability mechanism and a plausible incentive system to form a health service structure, which can guarantee the national welfare of public hospitals (21). Cao et al. (22) argued that hospitals should separate the management and administration system, reform the performance appraisal system, and strengthen the capabilities of primary medical resources such that the tiered diagnosis and treatment system can be fully formed (23). To break the profit mechanism of “feed doctors with drugs,” Ye et al. (24) considered that the multiple compensation system of public hospitals needs to be implemented. Furthermore, scholars believed that public hospital reform could improve the level of medical services and optimize the distribution of medical resources, thus increasing the accessibility and equity of medical care and promoting the improvement of public health (25). The Affordable Care Act, similar to the concept of “new medical reform” in China, has also made substantial contributions to expanding medical insurance coverage and lowering the mortality rate of the population (26, 27).

Public health is the fundamental measurement of people's wellbeing and economic development, and it is of great significance for production. A review of the existing literature shows that scholars have studied public health and public hospital reform from multiple perspectives, including the macro perspective and the micro perspective, and have made considerable research findings, setting a solid foundation for this study. Previous studies have argued that the factors which influence public health are not only individual participation but also government involvement; however, few studies have examined the impact of external policy shocks as a variable on health. Similarly, while numerous scholars have affirmed the achievements of public hospital reform on questions such as resource allocation and service levels, little attention has been paid to the impact of reform on public health, and only some literature has analyzed the relationship between improvements in health services and public health. Therefore, the impact of public hospital reform as an exogenous variable shock on public health is a question that needs to be answered urgently.

Based on the above questions, this study explored the impact of public hospital reform on public health. The empirical results showed that comprehensive public hospital reform significantly improves the level of public health, the indirect effect of the reform through the increase of financial expenditures on medical care is not as effective as expected, and the promotional effect of the reform on the level of public health shows significant heterogeneity among regions. The marginal contributions of this study are mainly in three aspects. First, regarding the research perspective, previous studies were primarily limited to the provincial level, while the research focus of this study is at a national level and considers the basic implementation of public hospital reform from batch piloting to full roll-out, which makes up for the absence of research in this area to a certain extent. Second, unlike the qualitative analysis method used in previous literature, this study adopted a time-varying DID empirical analysis, which is conducive to overcoming the endogeneity problem arising from model construction and can exclude as much as possible the effects caused by interfering factors other than the reform policy so as to estimate the unbiased net policy effect and reveal the effect of public hospital reform on the public health more comprehensively. Third, based on the fact that China's regional development is imbalanced, the extent of the impact of public hospital reform on public health in the eastern, central, and western regions of China are explored separately, thus making the study more consistent with the reality of China's vast territory and providing policymakers with a better perspective for decision-making.

## 3. Policy evolution and research hypothesis

### 3.1. Policy evolution

Even after the founding of New China, people's physical health was generally poor, and their demand for medical care could not be satisfied. Due to this lack of improvement in the healthcare sector, the country proposed to institute a three-tier medical diagnosis and treatment system to ensure the fairness and accessibility of the nationwide medical system. Since then, the labor insurance system and the publicly funded health system have also come into being, which significantly contributed to the development of healthcare in China and basically met people's medical needs. In the early years of the country, public hospitals aimed at general interest and national welfare greatly safeguarded people's right to life and health and fully embodied the government's original intention of working for the wellbeing of the people. The publicly funded health system largely maintained social stability and economic reconstruction for more than 30 years and became one of the most comprehensive medical systems in the world at that time.

In 1978, China officially kicked off the prelude to reform and opening up. Its fundamental goals, improving productivity and promoting economic growth, not only changed the path of the economic sector's development but also influenced the direction of the non-economic sector. The government's gradual withdrawal from the healthcare sector induced the medical system to shift toward a market-oriented path. In the early phase of the reform and opening up, marketization overcame many shortcomings of the public hospitals, such as a lack of competition and enthusiasm, a rigid structure, and poor service efficiency. It encouraged the innovation of public hospitals' management, salary subsidies, and other aspects in a short period and met the diversified medical needs of the people. However, with the government's financial support unable to maintain the regular operation of public hospitals, the profit motive gradually strengthened. Eventually, the loss of national welfare in public hospitals became more apparent and problematic, such as the supply-side induced demand, “high price and poor quality” of medical services, and the intensive relationship between doctors and patients. To resolve this chaotic phenomenon, in 2010, China proposed in the *Opinions* to form a more scientific and standardized public hospital management system. Through the government's financial assistance, the profit-seeking path of public hospitals and the “feeds doctor with drugs” situation were to be eradicated. A reasonable regulatory framework, operational system, and “white angel” image of the doctors were to be established. Hence, the development of public hospitals was to be rectified and the basic healthcare needs of the population were to be satisfied.

In 2010, the Ministry of Health launched public hospital reform to solve these problems. The reform can be separated into three parts. In the first part on management, the document emphasized that the modernization of hospital management must be undertaken and the decisions made by hospitals must be guided by public welfare and supervised by multiple channels. The second part focused on minimizing patient costs, particularly aimed at reducing the cost of medicines and medical consumables, such that the doctor could no longer benefit from prescribing medicines. To narrow the gap between urban and rural medical situations, the third part called for replanning the layout of public hospitals. More medical resources were to be allocated to rural hospitals, and the tier diagnosis service model needed to be established. To implement public hospital reform smoothly, the government selected 16 cities, including Anshan City, Shanghai City, Xiamen City, and Ezhou City, as national pilot cities to explore effective paths. In 2014, 17 cities, including Tianjin City, Ningbo City, Tangshan City, and Liuzhou City were selected as the second batch of pilot cities to carry out reform work. In 2015, the government assigned 66 cities, including Nanjing City, Xuzhou City, and Wenzhou City, to implement the third batch of reform. In May 2015, the government issued the “Guidance on the Comprehensive Reform Pilot of Urban Public Hospital,” which focused on breaking the profit-seeking mechanism of public hospitals and required all departments to jointly continue to do a good job in the next comprehensive reform. The fourth batch was formally implemented in 2016, with 100 pilot cities, including Haikou City, Zhongshan City, and Yueyang City. In 2021, the government again issued the *Opinions on Promoting the High-Quality Development of Public Hospitals*, which proposed that the next stage should adhere to people's health, be government-led, be focused on national welfare, and establish a modern hospital management system. In this way, public health and healthcare infrastructure were to be improved more comprehensively. To date, public hospital reform has been implemented for more than 10 years, and the capacity of China's medical service has significantly improved.

### 3.2. Research hypothesis

Improving public health is the fundamental goal of public hospital reform. The *Opinions* clearly emphasize the need to improve the public hospital's service system and management structure. It also proposed to augment the medical security payment by deepening the personnel reform and increasing government financial investment in health. We observed that this reform aimed to bring public hospitals back to the goal of national welfare from many perspectives, such as the reasonable mobilization of human capital, the continuous improvement of medical facilities, the innovation of management, the breaking up of the situation of medicine to support medical care, and other strong measures. The general public could, therefore, “dare to see a doctor,” have more equitable access to medical and health services, and improve their physical wellness. Therefore, the public hospital reform, in theory, was a significant step forward in improving public health.

Hypothesis 1: The reform of public hospitals will positively affect public health.

As an essential part of this reform, increasing the government's financial investment in health is the key to breaking the situation of public hospitals being “fed by medicines.” On the one hand, government financial investment can reduce the revenue channels of public hospitals to service fees and government subsidies but not drug revenues. It can also promote market-based pricing of medical services and establish a service price system that reflects the value of services and technical labor, so the welfare of patients and doctors can be enhanced. Using a doctor-patient relationship model, Chen et al. (28) found that the elimination of drug surcharges not only benefited the quality and supply of medical resources but was also able to reduce the cost of patients. On the other hand, Kou (29) argued that to deal with public health problems in China, it is necessary to rationalize the use of health resources and improve the efficiency of medical services so that social support and healthcare systems can be effectively used. In addition, the increase in financial support can guarantee capital construction and equipment purchase. Research in critical areas, personnel training, and even retiree pension will all be satisfied soundly. With the government's financial support, the service system of medical institutions can be built jointly, and the distribution of medical resources can be handled with necessary approvals. Eventually, public health will be improved.

Hypothesis 2: Public hospital reform affects the level of public health by increasing the government's financial investment in health.

Compared with the central-western regions, the eastern region has a better economic foundation to produce a higher health environment, such as a higher degree of marketization, relatively abundant medical resources, more comprehensive public health services, and a relatively high level of human capital. In the meantime, with the transformation and upgrading of the industrial structure in the eastern region, the negative environmental externalities brought by economic development are decreasing. Therefore, the level of public health in the eastern region is high. However, in central-western regions, with the scarcity of medical resources and the deficiency of infrastructure facilities, the supply of education and medical resources can't catch up with the growing demand for health services from the population. Therefore, theoretically, the impact of public hospital reform on public health in the Chinese regions will exhibit significant differences.

Hypothesis 3: The impact of public hospital reform on public health is regionally heterogeneous.

## 4. Data sources, variables, and model

### 4.1. Model selection

This study used a quasi-natural experiment, the public hospital reform that began in 2010, to examine its impact on public health levels. The method used in this study is a frontier research method in economics: the time-varying difference in difference model, intending to obtain plausible causal inferences. The main rationale of this method is to identify the net effects of policy shocks by comparing the policy effects of the treatment group and the control group before and after the policy shock based on the construction of counterfactual events. Specifically, this study took the time dimension as a first difference to compare the effects of public hospital reform before and after. Second, to examine the differences between cities that carried out public hospital reform and other cities, the study used the regional dimension as the second difference. Referring to Wang et al. (30) and Guo et al. (31), this study constructed an econometric model as follows.


(1) $$\begin{array}{l} y_{ct}=\;\alpha +\;\beta_{1}DID_{ct}+\;\varphi X_{ct}+\;\gamma_{t}+\;\sigma_{c}+\;\varepsilon_{ct}, \end{array}$$

where *y*_*ct*_ is the explained variable, representing the health level of the population in a given city *c* at the time of year *t*; *DID*_*ct*_ is the core explanatory variable, representing whether the city is under implementation or not; *X*_*ct*_ represents the control variables that change with city *c* or year *t* and affect public health, including the urbanization level, the education level, government scale, environmental pollution, and openness level; σ_*c*_ represents city-fixed effects, controlling all city-level factors that do not change over regions, such as geographical location; γ_*t*_ represents a time-fixed effect, controlling time-level features that do not vary from time to time, such as macroeconomic changes; and ε_*ct*_ indicates the error term. The core explanatory variable *DID*_*ct*_ estimated coefficient β_1_ is the main focus of this study, which reveals the effect of public hospital reform on public health compared with non-implemented cities.

### 4.2. Variables description

#### 4.2.1. Explained variable

Public health. Generally, public health is influenced by many complex factors such as ecological environment, economic conditions, education level, lifestyle, social factors, and medical level; therefore, it is difficult to find a multi-dimensional proxy indicator. Most of the indicators, such as population mortality, life expectancy, and infant or maternal mortality, have been used in past studies to measure public health. However, since it is difficult to distinguish structural differences in public health from macro data, based on the definition of health by the World Health Organization and with reference to previous studies, this study used population mortality rate (*PMR*) to measure the public health level in the locality. In general, a lower population mortality rate represents a higher level of public health. In addition, to analyze the mechanism of the effect of public hospital reform on public health, the article adopted the per capita medical fiscal expenditures in cities (*lnmedical*) as a proxy variable to measure the governmental financial investment in health.

#### 4.2.2. Explanatory variable

*DID*_*ct*_. Since the public hospital reform cities are implemented in four phases, this study adopted a time-varying DID model to evaluate the policy of public hospital reform in cities. *DID*_*ct*_ is defined as follows: if the city is in the year of policy pilot implementation or subsequent years, then *DID*_*ct*_ = 1; otherwise *DID*_*ct*_ = 0, thus forming a treatment group and a control group.

#### 4.2.3. Control variables

To obtain objective estimates of policy effects, this study controlled for variables that varied over time or regions and may affect public health. With reference to previous studies, the following control variables were selected: (1) urbanization rate (*urb*), expressed as the ratio of the resident urban population to the total population; (2) education level (*edu*), expressed as the number of college students per 10,000 people; (3) government scale (*gov*), is replaced by the annual government revenue; and (4) environmental pollution level (*fog*), which is substituted by the annual air emissions of each city. A large number of studies have shown that environmental problems have a negative impact on the health level of residents. The spillover and exogenous determinacy of air pollutants are stronger compared with other pollutants. This study adopted the annual emissions of smoke and dust as a proxy for the level of air pollution, referring to Sun et al. (32). (5) Level of openness (*fdi*) was represented by the total foreign direct investment in the current year. To avoid bias such as heteroskedasticity due to excessive numerical difference, the control variables in this study are all processed logarithmically.

### 4.3. Data sources and description statistics

In this study, panel data from 269 prefecture-level cities in China from 2008 to 2019 were as the research sample to evaluate the policy effects of the reform of public hospitals. According to the availability and validity of data, data from some cities such as Tibet, Turpan, and Haidong were removed from this article. All data in the article were acquired from the City Statistical Yearbook, Regional Statistical Yearbook, and EPS database, and some missing data were made up by manually collecting annual reports from each prefecture-level city. The data processing and empirical regression were mainly done through Stata16. Table 1 presents the descriptive statistics of each continuous variable.

**Table 1:** Descriptive statistics for the variables.

**Var**	**Obs**	**Mean**	**Std. dev**.	**Min**	**Max**
PMR	3,228	6.340	2.148	0.790	23.100
Lnmedical	3,228	6.263	0.653	4.150	9.050
Lnurb	3,228	3.918	0.299	2.854	4.605
Lnedu	3,228	4.571	1.160	0.000	7.675
Lngov	3„227	13.750	1.134	10.174	18.087
Lnfog	3,132	9.648	1.152	3.526	15.458
Lnfdi	3,131	9.950	1.883	1.099	14.941

## 5. Empirical analysis

### 5.1. Results of DID

In this study, we constructed an empirical model based on equation (1) and gradually added control variables for regression. Table 2 gives the regression results of the impact of the public hospital reform on public health. In column (1), the estimated coefficient of the reform indicated a significant negative relationship between public hospital reform and population mortality rate and based on the explanatory variable's meaning: the lower the population mortality rate is, the higher public health will be, which means that it can improve public health as well, hence verifying hypothesis 1. After gradually adding control variables, the significance of the coefficient did not change. Still, the net effect of the policy did alter, suggesting that the control variables did affect the population mortality rate. Overall, public hospital reform can significantly reduce the population mortality rate and promote public health.

**Table 2:** DID regression results.

**VAR**	**PMR (1)**	**PMR (2)**	**PMR (3)**	**PMR (4)**	**PMR (5)**	**PMR (6)**
DID	−0.438^***^ (0.135)	−0.439^***^ (0.134)	−0.437^***^ (0.134)	−0.429^***^ (0.133)	−0.452^***^ (0.133)	−0.498^***^ (0.136)
Lnurb		−1.336^***^ (0.418)	−1.261^***^ (0.426)	−0.928^**^ (0.416)	−0.972^**^ (0.416)	−1.087^**^ (0.426)
Lnedu			−0.152^**^ (0.071)	−0.162^**^ (0.072)	−0.166^**^ (0.074)	−0.167^**^ (0.075)
Lngov				−0.414^**^ (0.166)	−0.464^***^ (0.173)	−0.534^**^ (0.217)
Lnfog					−0.064 (0.046)	−0.062 (0.048)
Lnfdi						0.063 (0.061)
Con	5.724^***^ (0.070)	10.772^***^ (1.577)	11.133^***^ (1.575)	15.240^***^ (2.396)	16.699^***^ (2.553)	17.467^***^ (2.895)
Obs	3,228	3,228	3,228	3,227	3,132	3,042
*R* ^2^	0.205	0.207	0.208	0.209	0.212	0.219
Year-fix	YES	YES	YES	YES	YES	YES
City-fix	YES	YES	YES	YES	YES	YES

***, **, and * represent statistical significance at the 1%, 5%, and 10% levels, respectively. The values in parentheses are robust standard errors for clustering to the city level.

Further, from Table 2, it can be found that the urbanization rate has the largest negative effect on population mortality, concluding that urbanization effectively improves public health. The possible reason is that urbanization can bring more medical sources and more income for residents and can exaggerate the scale effects of infrastructure, and with the improvement of these factors, resident health will also be promoted. The marginal impact of government scale and education level on population mortality is also statistically significant and negative, improving public health, validating the results of Grossman (5). While the coefficient of the openness and environmental pollution, although not statistically significant, has a positive effect on population mortality, the possible explanation is that due to the lack of environmental regulations in China, as the openness increases, it attracts low-quality foreign investment in pollution-intensive industries, which in turn exposes the regional environment to a low level of equilibrium and creates a “pollution sanctuary.” This leads to an increase in population mortality.

### 5.2. Robustness tests

#### 5.2.1. Parallel trend test

According to the above study, the public hospital reform has effectively reduced the population mortality rate and thus improved public health. However, as a model for policy effect assessment, the unbiased estimation of time-varying DID results was needed to satisfy a prerequisite assumption that the treatment and control groups must have the same or similar development trend before the policy implementation so that the influence of other possible variables can be excluded. Otherwise, the estimation results were to be biased. To verify the feasibility of parallel trends and further observe the dynamic effects of the policy, this study referred to Jacobson et al. (33) and Guo et al. (34) and adopted the event analysis method to study the dynamic effects of the reform. The following regression model is based on Equation (1).


(2) $$\begin{array}{c} y_{ct}=\;\alpha +\;\overset{2}{\underset{j=-6}{\sum }}\beta_{j}D_{c,\;t-j}+\;\varphi X_{ct}+\;\gamma_{t}+\;\sigma_{c}+\;\varepsilon_{ct} \end{array}$$

In the equation, *D* is a dummy variable and takes the value of 1 if the city *c* implements the reform in year *t* − *j*; otherwise, it is 0. β_0_ represents the policy effect in the current period of the reform, β_−6_ to β_−1_ represents the policy effect before the reform, and β_1_ to β_2_ represents the after.

**Figure 1:**
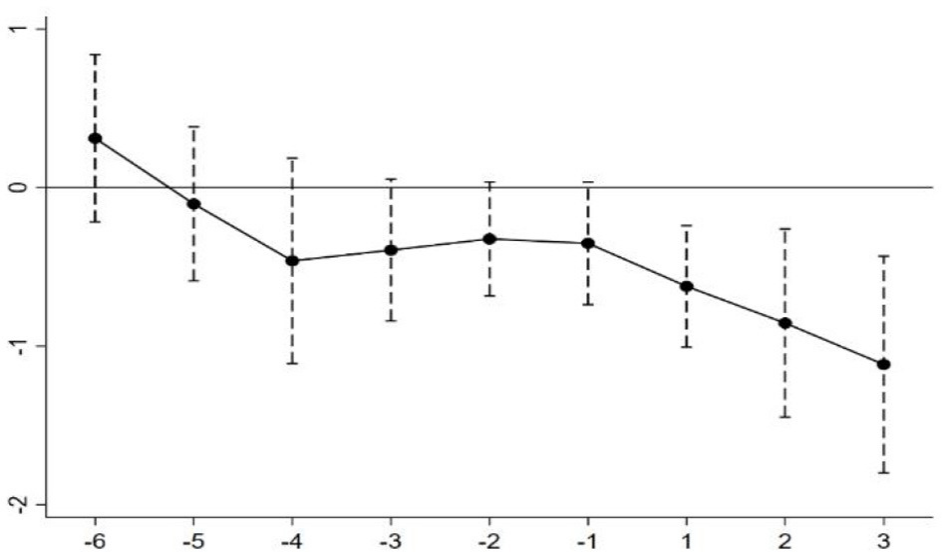
Parallel trend test.

Figure 1 demonstrates the coefficient of mortality rate. The dots represent the values of the coefficients, and the dashed lines represent their 95% confidence interval. As can be seen from Figure 1, the estimated coefficients basically fluctuated around zero before the policy shock, and none of them could pass the 95% confidence interval. It implies that the difference in development between treatment and control groups before the policy shock was not significant, validating the premise of a parallel trend.

It should also be noted that the estimated coefficients have a significant downward trend in the year of the reform implementation and have the momentum of decreasing annually, which indicates that the reform of public hospitals can effectively reduce the mortality rate, and the policy effect will increase with time.

#### 5.2.2. Tailoring test

Considering that the estimation results may be affected by extreme values, which may generate estimation being biased, in this study, all the continuous variables were treated with bilateral tail reduction at 5% and 95%, respectively, and reoperated the model. The results are shown in Table 3. The results showed that there were no significant changes in the regression coefficient and remained negative even after excluding the extreme values, thus verifying the robustness of the above study.

**Table 3:** Results of robustness tests.

**VAR**	**(1) Pre 3**	**(2) Pre 1**	**(3) Tailoring**
DID	0.127 (0.115)	0.074 (0.109)	−0.450^***^ (0.119)
Constant	17.817^***^ (2.896)	17.758^***^ (2.903)	14.144^***^ (2.662)
Observations	3,042	3,042	3,042
*R* ^2^	0.215	0.215	0.220
Controls	YES	YES	YES
Year-fix	YES	YES	YES
City-fix	YES	YES	YES

***, **, and * represent statistical significance at the 1%, 5%, and 10% levels respectively. The values in parentheses are robust standard errors for clustering to the city level.

#### 5.2.3. Setting virtual time

To further avoid biased regression results, we referred to Fan and Tian (35) to construct a virtual policy implementation so that we could examine whether the changes in the explained variable are related to the implementation of the policy. In this study, we assumed that the implementing year of the reform is uniformly advanced by 1 year and 3 years and re-regressed to determine whether the coefficients of their explanatory variable are statistically significant. The results are listed in Table 3, which shows that the estimated coefficient of the explanatory variable is not significant in the DID model. Table 3 indicates that the difference in public health between the implemented and non-implemented cities is due to the “dividends” brought by the reform. The robustness of the baseline regression results was further verified.

#### 5.2.4. Placebo test

**Figure 2:**
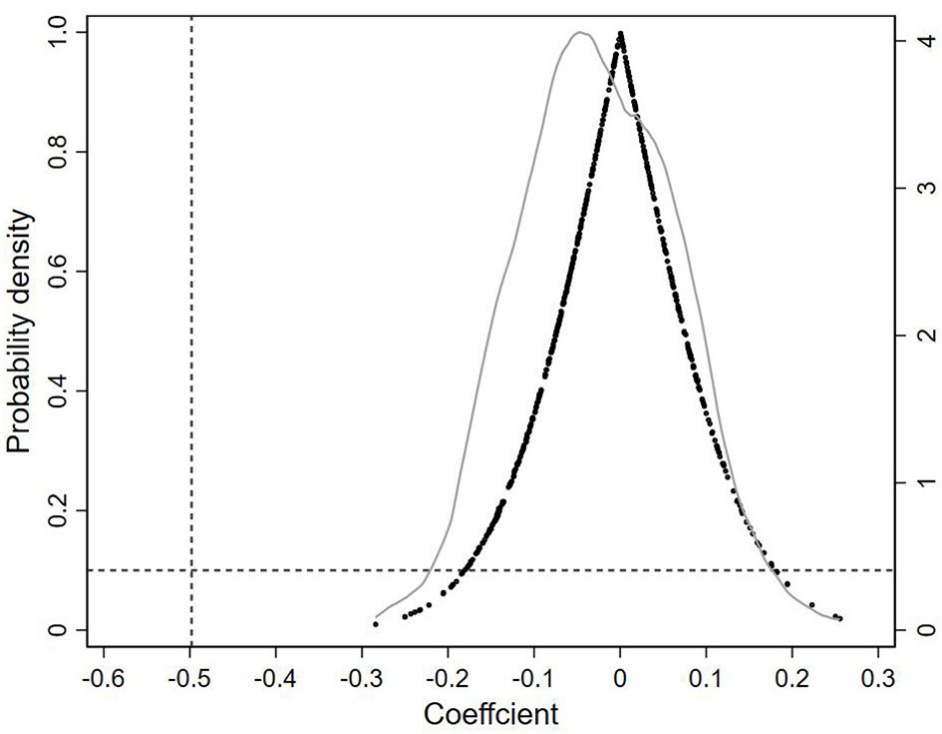
Results of the kernel density distribution of the DID of the placebo test.

In the meantime, this study referred to Guo et al. (36) to conduct placebo tests by constructing a fake treatment group. If the coefficient remained significantly negative, it indicated that the changes in population mortality rate were not driven by the reform but by other unobserved factors, and vice versa, supporting the robustness of the result. In this study, a random sample with put-back was conducted from the sample of 269 cities, keeping other control variables fixed, and then a DID regression was performed. To enhance the convincingness of the estimation results, this study repeated the sampling 500 times, and the estimated coefficients of the reform are shown in Figure 2.

Each black dot in Figure 2 represents the estimated coefficients of the explanatory variable in the false treatment group. The results showed that the estimated coefficients were mainly distributed around the value of 0 and were close to the normal distribution. The position of the vertical line in Figure 2 indicates the estimated coefficient of the baseline regression (i.e., −0.498), which falls at the end of the random sample distribution, is much smaller than the estimated coefficients of the placebo. It indicates that the population mortality rate is not significantly affected by the reform under the placebo treatment group, confirming that the reduction in mortality rate was indeed a result of the reform.

### 5.3. Results of the mediating effect

The empirical results from the benchmark regression indicated that public hospital reform improved public health by lowering the population mortality rate. However, the channels through which the reform goals are achieved need to be further analyzed. The construction of empirical models needs to be based on the reform programs. In the *Opinions*, it is clearly stated that the reform focuses on fundamental changes in the income-generating mechanism of doctors. The public hospitals should eliminate their profit-seeking character, highlight the “national welfare” orientation, and achieve the reform goals by increasing financial support and enhancing the government's responsibility in the healthcare department. Therefore, the support of the government's financial investment in health is an important path to achieving the goal. This study used the per capita medical fiscal expenditures as a proxy for the strength of financial investment to test the transmission path of the reform on public health.

Based on equation (1), this study first explored the effect of the mediating variable (*lnmedical*) on the explanatory variables to verify whether there was a significant linear relationship between them. Second, based on equation (1), the mediating variable was added to test the effect of financial expenditure on the results of the benchmark study. The model is as follows.


(3) $$\begin{array}{c} \text{lnmedical}=\;\alpha +\;\beta_{2}DID_{ct}+\;\varphi X_{ct}+\;\gamma_{t}+\;\sigma_{c}+\;\varepsilon_{ct} \end{array}$$

(4) $$\begin{array}{c} y_{ct}=\;\alpha +\;\beta_{3}DID+\;\theta lnmedical+\varphi X_{ct}+\;\gamma_{t}+\;\sigma_{c}+\;\varepsilon_{ct} \end{array}$$

The bootstrap method referred to Wen et al. (37) and Chen et al. (38) to test the mediation effect. First, there were 500 random replicates according to the samples that were put back, which were then used to estimate the mediation effects; if their confidence intervals of 90% or above did not include zero, it means that the original hypothesis *H*_0_ = β_2_ × θ was rejected. If the coefficients were statistically significant, the mediation effect was valid.

The regression results of the mediating effect under the Bootstrap method are given in Table 4. The outcome showed that the mediating effect of expenditure holds significantly for the population mortality rate, and its direct and indirect effects did not include zero at the 90% confidence interval. Thus, hypothesis 2 was verified. However, the impact of reform on the population mortality rate through the mediating channel was much lower than its direct effect (indirect effect = −0.020; direct effect = −0.434), which may be explained by the imperfection of supporting policies such as management system, staff deployment, remuneration mechanism, and promotion mechanism during the reform process in cities. These issues were the result of the lack of clarity on the responsibility between the government departments and public hospitals and led to the inability to fully utilize the government's financial support for public hospitals.

**Table 4:** Mediating test.

	**Observed coef**.	**Std. err**.	**z**	***p* > |z|**	**Normal-based [90% conf. interval]**	
Indirect	−0.020	0.011	−1.768	0.078	−0.038	−0.001
Direct	−0.434	0.144	−3.014	0.003	−0.670	−0.197

### 5.4. Regional heterogeneity test

As a typical large economy, China has an obvious imbalance in regional development, such as the basic healthcare and urbanization ratio among regions. To analyze the regional differences in the impact of the reform on public health, the study divided China into three major regions: eastern, central, and western. Then, we estimated the impact of the reform separately. The results are shown in Table 5.

**Table 5:** Regional heterogeneity test.

**VAR**	**(1) Eastern**	**(2) Central**	**(3) Western**
DID	−0.207 (0.213)	−0.603^**^ (0.249)	−0.687^***^ (0.241)
Constant	32.939^***^ (5.548)	11.505^*^ (5.842)	21.507^***^ (4.653)
Observations	1,093	1,069	880
*R* ^2^	0.332	0.204	0.163
Controls	YES	YES	YES
Year-fix	YES	YES	YES
City-fix	YES	YES	YES

***, **, and * represent statistical significance at the 1%, 5%, and 10% levels, respectively. The values in parentheses are robust standard errors for clustering to the city level.

After the year-fix and time-fix effects were under-controlled, the estimated coefficient of the reform in the eastern region was not statistically significant (refer to Table 5). At the same time, the coefficient in the central and western regions were −0.603 and −0.687, respectively. The coefficients were not only significant at the 1% level but also much smaller than that of the eastern region. This indicates that the reform in the central and western regions could suppress mortality more significantly and promote public health, which is consistent with the benchmark estimation results at the national level, thus verifying hypothesis 3. The above comparison shows that the positive effect of public hospital reform on public health was significantly stronger in the central and western regions than in the eastern region. The probable reason is that the eastern region has more advantages than the other two regions. For instance, economic base, marketization, medical resource allocation, public health services, residents' human capital, government financial scale, and other health-influencing factors. At the same time, with the transformation and upgrading of the industrial structure in the eastern region, the tertiary industry has been increasing in proportion to the GDP, and the improvement of environment; therefore, the reform to break the profit-seeking layout, improve the management system, and a series of policies brought about by the eastern population has less impact. In contrast, urban medical resources in the central and western regions are relatively scarce, public infrastructure needs to be strengthened, and the supplement of education and medical resources is slower than the population growth rate. Moreover, the central and western regions have begun to gradually take over the role of manufacturing industries from the eastern coastal areas. With the secondary industries accounting for an increasingly high proportion and the ecological environment deteriorating, the positive effect of reform on the level of public health has been significant.

## 6. Conclusion and implications

Compared with the qualitative analysis of public hospital reform in previous literature, this study, using the panel data of 269 prefecture-level cities from 2008 to 2019, examined the net effect of public hospital reform on public health and conducted relevant robustness tests by applying the time-varying DID model. Further, we identified the mechanism of the impact and analyzed the heterogeneity of the policy effects of public hospital reform.

The following conclusions were obtained: first, at the national level, public hospital reform significantly contributed to public health. Specifically, compared with the non-implemented cities, the reform can effectively reduce the rate of mortality by 0.498 per thousand of the population. The result showed that the policy is an important driving strategy to improve public health and validates previous qualitative studies from the perspective of empirical models. Second, the mediating result showed that the reform can improve public health by increasing government medical expenditure. Still, the indirect effect was minimal compared with the direct impact; in fact, the proportion of indirect effect was only 4.405%. It showed that the role of medical expenditure has not been fully utilized in the reform process. Third, there was heterogeneity in the impact of reform in different regions, it significantly promoted public health in the central and western regions, but not in the eastern regions. This indicated that the effect of reform policy on the progress of public health varied from region to region, and the impact of the reform policy was more significant in regions with weaker economic strength and poorer ecological environments.

Based on the above research findings, this study puts forward the following policy recommendations: first, public hospital reform is an important strategy to improve public health. Governments at all levels should ensure the orderly implementation of the reform and expedite the formation of medical service systems that guarantees the national welfare of public hospitals. The path of this policy can lay a solid foundation for further public hospital reform. Second, positive actions need to be taken to optimize the structure of financial expenditures, implement the responsibility of healthy financial investment, and accelerate local economic development to enrich the financial reservoir. The central government should increase its ability to make financial transfers to the provinces and improve the efficiency of health financial investment to establish a long-term health financial investment that is in line with people's health needs. Third, there are certain differences in the economic, social, and ecological conditions of different regions in China, and the “one-fits-all” approach cannot meet the needs for high-quality development of local health. The governments at various levels need to customize the policies according to local conditions to promote balanced development in each region, accelerate the process of building *Health China*, and continue to provide health protection for China's high-quality development.

There are still some limitations that could be studied further. On the one hand, due to data limitations, this study used the population mortality rate as a proxy for general health rather than directly measuring public health levels. On the other hand, we only used one of the measurements as a transmission mechanism to study. However, with a specific focus on public hospital reform, the research is still worthy of attention. We believe that further work will provide useful supplements in these aspects.

## Data availability statement

The raw data supporting the conclusions of this article will be made available by the authors, without undue reservation.

## Author contributions

BG and WF designed the study, performed the research, analyzed data, and wrote the paper. HC collected most of data. JL conducted empirical analysis.
